# Cognition does not automatically influence perception: Evidence from neural encoding of colors belonging to different categories

**DOI:** 10.1073/pnas.2538139123

**Published:** 2026-06-09

**Authors:** Jasna Martinovic, Alexey A. Delov, Jana Tomastikova, Joel T. Martin, Galina V. Paramei, Yulia A. Griber

**Affiliations:** ^a^ Department of Psychology, School of Philosophy, Psychology and Language Sciences, University of Edinburgh, Edinburgh EH8 9JZ, United Kingdom; ^b^ Department of Sociology and Philosophy, Smolensk State University, Smolensk 214000, Russia; ^c^ School of Psychology, Liverpool Hope University, Liverpool L16 9JD, United Kingdom

**Keywords:** color, cognition, categorical perception, EEG, Whorfianism

## Abstract

The firmest evidence in favor of models that posit early high-level influences of cognition on perception comes from electroencephalography (EEG). Enhanced early, preattentive processing of light and dark blue feature changes compared to light and dark green changes was reported in Greek speakers, who have two basic terms for “blue” (*ghalazio/ble*). In the present three-experiment study, we systematically reevaluate this evidence and test an alternative model that the early difference waves in the human EEG instead mainly reflect contrast adaptation phenomena. We use the same classical oddball paradigm presenting alternating standards and deviants that systematically differ in color and/or luminance and chromatic contrast. We then calculate the visual mismatch negativity (vMMN), a putative index of preattentive feature change processing and predictive coding derived from EEG data, by subtracting the activity elicited by standards from that elicited by task-irrelevant deviants. Our experiments demonstrate the following: 1) vMMN is driven by contrast adaptation, being observable only in the presence of contrast differences between the stimuli and not reliably observed for categorically different hues equated in contrast; 2) there is no reliable difference between green- and blue-related difference waves in speakers (Russian) with two basic blue color categories, the difference waves, again, being driven by contrast rather than their categorical content. Our findings are highly significant for the debate concerning the interface between perception and cognition, as the absence of early categorical effects speaks against models that predict Whorfian-type preattentive cognitive influences on perception.

## Significance

The Whorfian hypothesis posits that basic language categories alter one’s perception of the world in a fundamental manner. Some of the most compelling evidence in favor of this hypothesis came from electrophysiological responses that indicated early differences between speakers of languages with different numbers of basic color categories. The brain response taken as an indicator of these differences was considered to be a robust marker of early, preattentive processing and predictive coding. In the current multiexperiment study, we present evidence that early electrophysiological differences to color reflect signatures of hue, saturation, and luminance contrast and adaptation to this contrast, rather than of linguistic categories. This means that evidence in favor of early, preattentive categorical color perception has been significantly eroded.

A key question in cognitive science is whether linguistic categories and concepts can directly shape perceptual processes, as proposed by the Whorfian hypothesis. The cognitive domain of color categories presents a particularly salient natural experiment for evaluating putative Whorfian effects, since some languages differ in how they categorically partition the color space (1–3). Furthermore, with its millisecond temporal resolution, electroencephalography (EEG) is a particularly informative tool for testing claims about early perceptual and cognitive processing. Thus, color category research—both behavioral (3, 4) and electrophysiological (5, 6)—has played an important role in the ongoing debate on the extent of Whorfian effects on color perception and the penetrability of perception by cognition more generally.

As discussed in the keynote dialog of Ahissar and Scholl on the topic of cognitive penetrability of perception at the European Conference of Visual Perception in 2017, the most robust and direct evidence in favor of penetrability remains the event-related potential (ERP) study by Thierry and colleagues on Greek “blues” (5). ERPs represent EEG responses time-locked to an event of interest, averaged across multiple trials to remove nonsystematic noise. In cognitive electrophysiology, different ERP components are taken as indices of different cognitive processes (7). The visual mismatch negativity (vMMN) is one such component—a negative deflection in the ERP difference wave peaking at about 150 to 240 ms when elicited by different colors (8). It is obtained by subtracting the event-related neural response driven by the repeated, standard stimuli from the event-related response to rare, deviant stimuli. The vMMN is assumed to index a mismatch between the built-up sensory representation of the standard stimulus and a rare deviant stimulus (9, 10). Thierry et al. (5) used a classical vMMN oddball paradigm and found greater vMMN resulting from light and dark blue series of standards and deviants compared to light and dark green series in Greek speakers, who have two basic terms for “blue” (*ghalazio/ble*).

Thierry et al.’s study is considered to provide strong evidence in favor of models that posit high-level, top–down categorical effects on early, preattentive processing of color (11) and goes against the more recent categorical facilitation model, which argues that effects of color categories on perceptual processing are late and mediated by attention (12, 13). Thierry et al.’s findings on automatic categorical effects on early brain activity in the absence of a task that specifically recruits language also go against models that propose linguistic categories do not come into play in nonlinguistic versions of very similar tasks (14). However, a recent critical review of EEG studies on color categorization between speakers of different languages identifies some major problems with this body of work and asks for a methodologically sound reevaluation of the evidence base (15). These concerns are further compounded by specific issues with validity of early electrophysiological markers of visual feature-change detection and their robustness to contrast-adaptation effects (16).

Since classical oddball tasks involve streams of repeated stimuli (standards) interspersed by rare stimuli (deviants), with each standard stimulus repetition, there is a build-up of neural adaptation. In ERPs, adaptation manifests as repetition suppression (16), whose effects on the early, sensory parts of the EEG waveform are separable from expectation effects (17). Stimulus repetition led to a more negative waveform in the period of early visual evoked activity—reducing the amplitude of the P1 component and increasing the amplitude of the subsequent N1 component (17). Since vMMN overlaps in time with the P1 and N1, it is particularly vulnerable to repetition suppression. In the auditory domain, rapid and stimulus-specific adaptation has already been proposed as the underlying mechanism for a similar, standard-stimulus driven build-up in early positivity in evoked activity, related to echoic memory traces (18) and considered as the foundation of predictive coding (9).

After experimentally controlling for the effects of adaptation, the vMMN could not be reliably observed in response to orientation, luminance contrast, phase, and spatial frequency features of Gabor patches (16). Instead, an early deviant-related positivity (~100 ms) was observed, which led to the proposal that, rather than the vMMN, this positive deflection might in fact be the neural marker of an early predictive processing of elementary visual feature values. The N1 component, which temporally coincides with the vMMN, exhibits both adaptation and response selectivity for color (19). If we identify expectation violations, rather than simple repetition suppression effects with the underlying categorical perception processes that color oddball tasks were intended to tap into, this poses a very concrete challenge to earlier EEG studies reliant on negative deflections such as the vMMN as an indicator of automatic, preattentive feature-change detection.

**Fig. 1.**
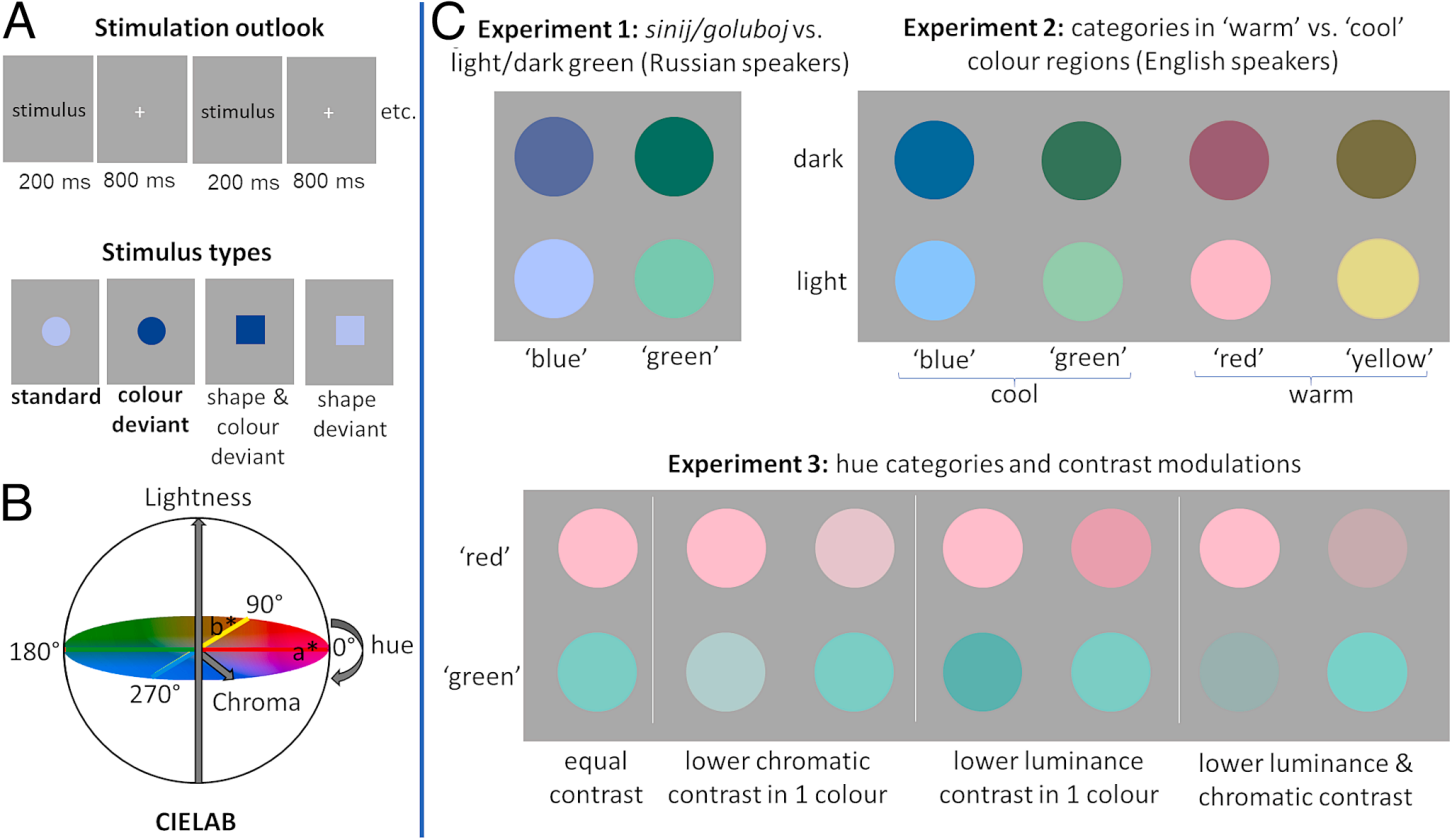
Experimental stimuli and the oddball paradigm. (*A*) Stimuli are presented in pseudorandom sequences of 3 to 5 standard stimuli of circular shape, followed by an oddball that is either a deviant only in colour, and thus task-irrelevant, or is also a target (i.e., square) shape. The example stimulus types are taken from a block in which light blue is the standard colour and dark blue the deviant colour. (*B*) CIELAB colour space, with lightness (L; range 0 to 100) as its vertical dimension, chroma (C; zero being achromatic) as the distance from the central lightness axis which defines the colourfulness of the stimulus, and hue as the angle of rotation on any of the horizontal planes within the space. Red and green are on the opposite ends of the 0 to 180° axis, labeled as a*, while blue and yellow lie along the 90 to 270° axis, labeled as b*. Stimulus colours from all experiments are plotted in CIELAB chromatic plane in *SI Appendix*, Fig. S1. (*C*) Examples of stimulus appearance in each experiment. Appearance of the stimuli is approximated rather than exactly reproduced by converting all colours to sRGB while ensuring that RGB values are scaled proportionately to fit within the standard screen gamut. Note increased chromaticness and a slight shift towards purple in the blue sample of Experiment 1, which replicates the samples used by Thierry et al. (5). Note also that in Experiment 3, red and green colours consistently belong to two different basic colour categories, but otherwise are either equal in chromatic and luminance contrast, different along one of these dimensions only, or different along both dimensions.

Considering the fundamental challenge to the validity of the vMMN as the index of early preattentive feature-change processing and the ongoing theoretical debates on the automaticity of categorical effects, it is important to reevaluate Thierry et al.’s findings and provide more robust evidence for theories of color cognition, and, more generally, for models of the perception/cognition interface. Here, we do that in three experiments, whose key aspects are depicted in Fig. 1:

(1) A close replication of Thierry et al.’s (5) paradigm conducted, in place of Greek speakers, with Russian speakers, who, too, have two basic terms for “blue” (*goluboj* “light blue,” *sinij* “dark blue”). The stimulus coordinates, oddball structure, and analysis approach closely followed the original study, while the language group differed.
(2) A conceptual extension with English speakers, with standards and deviants in the “cool” and “warm” regions of color space, respectively. For English speakers, the “cool” region includes two basic color terms (BCTs)—*blue* and *green*, whereas the “warm” region is lexically represented by four BCTs—*red, pink, yellow,* and *brown*. Pairs of light and dark blue and light and dark green belong to the *blue* and *green* BCTs, respectively; in comparison, light and dark shades in the red region should be labeled by distinct BCTs *pink* and *red*, and light and dark shades in the yellow region should be labeled by distinct BCTs *yellow* and *brown*.
(3) A final experiment in which we test whether the vMMN can be reliably observed for feature changes along three perceptual dimensions of color: hue, lightness, and saturation (colorfulness). If the vMMN is driven by a categorical distinction, it should be observed in all instances, as each block contains categorically distinct variations of “red” and “green.” Alternatively, if the vMMN depends on a co-occurring difference in color or luminance contrast, we should observe it only when contrast differences between “red” and “green” are introduced.

Foreshadowing our findings, we observe that EEG difference waves in the early time-window corresponding to the vMMN are driven by contrast adaptation, rather than by between-category differences. We fail to replicate Thierry et al.’s (5) observation of enhanced vMMN in speakers with two BCTs in the blue area of color space, their study’s outcome likely stemming from the fact that the original observation had an effect size whose 95% CI was very broad, indicating a less than precise estimation [μ_p_^2^ = 0.112, with a 95% CI of 0.004 to 00.273, estimated using the noncentral F distribution (20)]. Instead, we observe a series of robust, well-powered findings that consistently reflect effects of adaptation to luminance contrast, chromatic contrast, or both.

## Results

**Fig. 2.**
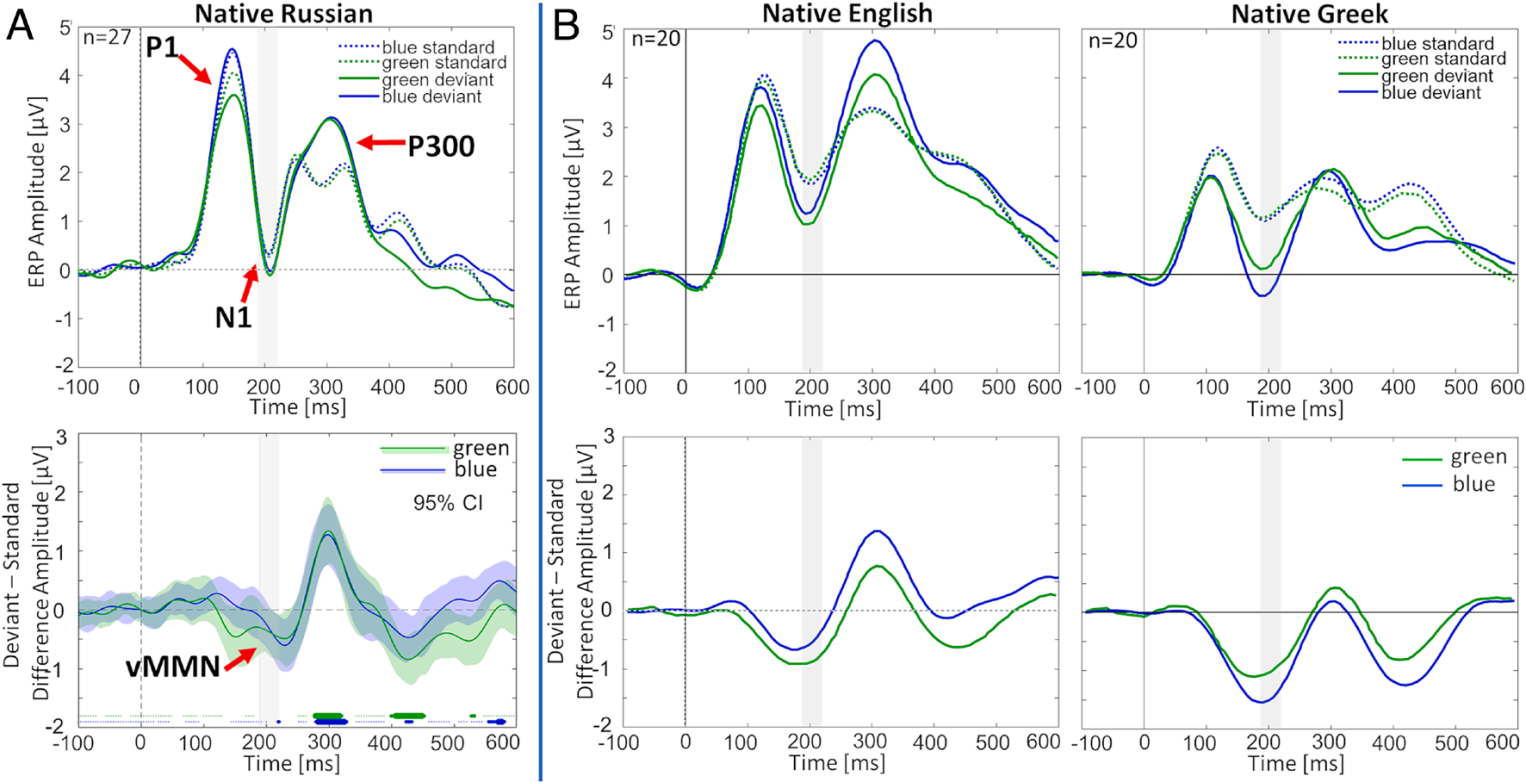
ERPs and difference waves from our sample of Russian speakers (panel *A*) and Thierry et al.’s samples of English and Greek speakers (panel *B*). Russian and Greek speakers are expected to exhibit a higher vMMN for blue due to two basic colour categories for dark and light blue, sinij/goluboj (Russian) or ble/ghalazio (Greek). However, the difference waves for “blue” and “green” in Russian speakers (panel *A*, *Bottom*) are highly similar and small, with little evidence of a robust difference against zero. The *Top* row depicts the grand-mean ERP waveforms elicited by green and blue standards and task-irrelevant deviants, which had identical colour coordinates across the two studies. EEG responses to task-relevant deviants (i.e., targets) are not shown, since they were explicitly attended and would, thus, not be useful in indexing automatic feature-change detection. The three most prominent ERP components in the stimulus-evoked response—P1, N1, and P300—are marked by red arrows and labels in panel *A*. The *Bottom* row depicts difference waves, created by subtraction of mean activity elicited by standards from that of identically coloured deviants. The vMMN is signposted by a red arrow and the label. In panel a’s depiction of the difference wave, Bayes factor (BF) values are indicated above the x -axis, with smallest dots indicating lack of significant differences from zero (BF < 0.33), medium-sized dots indicating moderate evidence for a significant difference (<3 BF < 10), and large dots indicating strong evidence of a deflection in the difference wave (BF > 10). The time-window of the N1 peak in our study is highlighted by a light gray overlay across all panels, to facilitate comparisons.

Fig. 2 depicts ERPs and difference waves from our Experiment 1 with Russian speakers, side by side with replotted waveforms from the Thierry et al. (5) study, digitized from their EEG figure (21). Our ERPs elicited by blue and green colors follow similar patterns to those for the English sample in Thierry et al. The difference waves also appear similar, with the vMMN occurring in roughly the same time-window, followed by a positive deflection which results from a higher P300 response for task-irrelevant color deviants. The P300 component reflects automatic attentional orienting (22), so it is unsurprising to see that it is higher for less frequent, task-irrelevant color oddballs. The major difference in the two studies’ findings is that we observe a vMMN that appears to be lower in amplitude, so that evidence for robust differences against zero remains limited (see Fig. 2 *A*, *Bottom* panel). This comparatively smaller vMMN also fails to exhibit any reliable differences between *sinij/goluboj* and light/dark green (BF_10_ = 0.301).

**Fig. 3.**
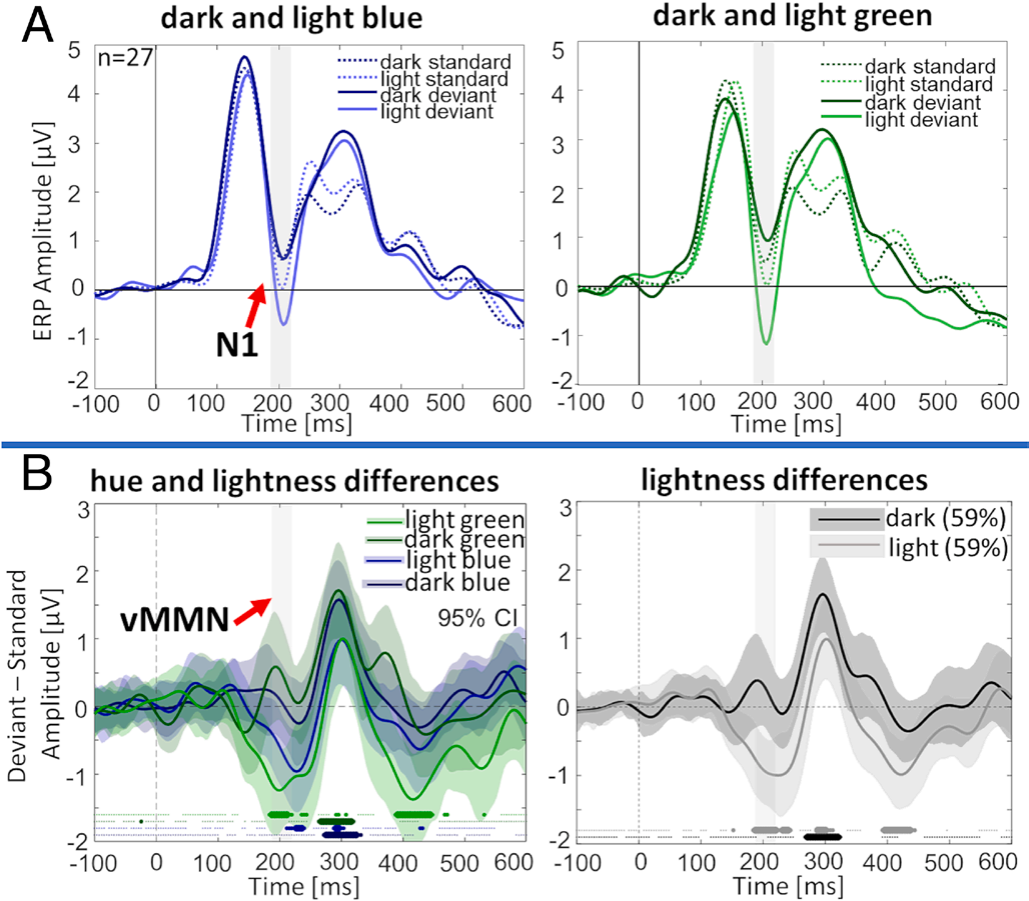
ERPs and difference waves from the experiment on Russian speakers, separated by lightness. (*A*) This panel depicts the grand-mean ERP waveforms elicited by light and dark standards and deviants for blue (*Left*) and green (*Right* panel). Note that during the windows characteristic of the N1 and vMMN components (shaded gray) it is the light standards and deviants that appear to elicit higher activity, and this is particularly the case with the light deviant, which would follow a set of repeated dark standards. (*B*) Difference waves, created by subtraction of light and dark standards from their respective deviants. The *Left* panel depicts the difference waves separately for each hue/lightness combination. The *Right* panel collapses across two hues to reveal differences by lightness alone. BF values are indicated above the x-axis, with smallest dots indicating lack of significant differences from zero (BF < 0.33), medium-sized dots indicating moderate evidence for a significant difference (3 < BF < 10) and large dots indicating strong evidence of a deflection in the difference wave (BF > 10). Note that averaging colours across lightness, which is depicted in Fig. 2*A*, conceals that for dark greens and blues, there is no robust vMMN present, with the component only being notable for light colours.

In order to further understand the factors that drive EEG amplitudes, it is useful to depict ERPs and difference waves separately for dark and light blues and greens (Fig. 3). This reveals that early evoked activity differs substantially between light and dark shades despite the fact that both color sets had the same luminance contrast against the background (59%). In fact, lightness-driven differences are more substantial than between-hue differences: Statistical analyses of ERP responses in the N1 time-window reveal a modulation only by lightness and deviance, which interact: Amplitudes are more negative for light deviants (−0.96 µV, 95%CI [(−1.33), (−0.60)], t(182) = 5.206, *P* < 0.001, 81% variance explained by the linear mixed effect model; for full model details, see *SI Appendix*, Supplementary Materials 1). For lighter colors, we see moderate to extremely strong evidence of a robust negativity in the difference wave (light blue BF_10_ = 4.491, light green BF_10_ = 218.993), but there is anecdotal to strong evidence of absence of a vMMN-type negativity for darker colors (dark blue BF_10_ = 0.444; dark green BF_10_ = 0.250). Bayesian *t* tests conducted across the time-course of the ERP (see *Bottom* panel of Fig. 2*A* as well as Fig. 3*B*) corroborate these statistical analyses.

**Fig. 4.**
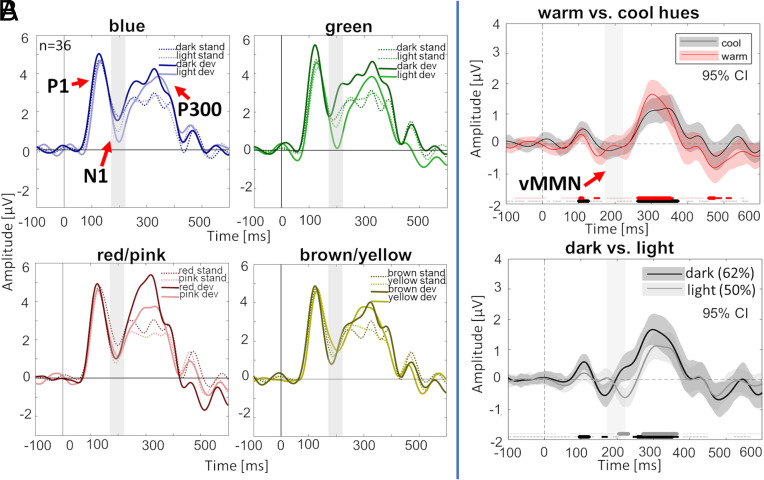
ERPs and difference waves from Experiment 2 with English speakers, who are expected to exhibit a higher vMMN for “warm” hues due to multiple basic colour categories denoted in that region of colour space (i.e., red/pink, or yellow/brown), as opposed to less categorically and lexically distinguishable “cool” region (green and blue). (*A*) This panel depicts the grand-mean waveforms elicited by light and dark standards and deviants for light/dark blue and light/dark green (*Top* panels), and red/pink and brown/yellow (*Bottom* panel). The overall waveforms are similar to those in Figs. 2 and 3, with P1, N1, and P300 components being most prominent. Note, however, the apparent differences in the latency of the N1 response in the gray-shaded window between dark and light versions of a colour (most pronounced for green and yellow), with the N1 peak appearing earlier for darker shades. (*B*) Difference waves, created by subtraction of warm and cool (*Top*) or light and dark (*Bottom* panel) standards from their respective deviants. BF values are indicated above the x-axis, with smallest dots indicating lack of significant differences from zero (BF < 0.33), medium-sized dots indicating moderate evidence for a significant difference (3 < BF < 10) and large dots indicating strong evidence of a deflection in the difference wave (BF > 10). Two points are of note: 1) The difference wave for warm and cool hues does not exhibit a significant deflection in the N1 window, but there appears to be an earlier positive deflection at ~110 ms, coinciding with the P1 window of the ERP; 2) As in Fig. 2*A*, averaging hues across lightness levels conceals differences between dark and light colours. We see evidence for a positive deflection at ~110 ms and an earlier subsequent vMMN for dark colours, which also happen to be higher in luminance contrast against the background. The effects of contrast on the vMMN for dark vs. light colours with an absence of a reliable effect of the category for warm vs. cool hues is, again, inconsistent with an interpretation of early evoked differences in the EEG as a signature of categorical processing. The time-window of the N1 and vMMN components is highlighted with a gray overlay.

In Experiment 2, conceptual replication with English speakers (Fig. 4; for Bayesian stats across the difference wave timecourse, see Fig. 4*B*), we fail to observe a reliable vMMN driven by “warm” (BF_10_ = 0.134) or “cool” colors (BF_10_ = 0.436), despite the fact that “warm” colors are denoted by two BCTs across their light and dark variants (*pink/red* or *yellow/brown*), unlike “cool” colors, light and dark green or blue, which are denoted by varied hyponyms or achromatic-modified terms (for names provided by our participants, see *SI Appendix*, Supplementary Materials 3). In Experiment 2, we introduced a small difference (12%) in luminance contrast between dark (62%) and light (50%) shades of each color. The luminance contrast difference is too small to alter categorical or relative (dark or light) belongingness but is expected to elicit different degrees of adaptation of ON (light) and OFF (dark) luminance mechanisms (23). In line with this experimental manipulation, we observe difference wave modulations by luminance contrast, with a significant vMMN for lighter (BF_10_ = 2.340) but not darker colors (BF_10_ = 0.142) in the 170 to 220 ms time-window. Fig. 4*B* reveals that this is due to a contrast-dependent shift in the latency of the vMMN component, with the vMMN appearing earlier for darker as opposed to lighter colors. For darker colors, the subsequent P300 deflection in the difference wave also appears earlier, resulting in the average amplitude being approximately zero in the N1/vMMN time-window. There is also an early positive component in the difference wave at ~110 ms, coinciding with the P1 component of the ERP, which is robustly present for darker colors only (BF_10_ = 2.03; light colors BF_10_ = 0.21; see also Fig. 4*B*). The enhanced P1 for darker colors is in line with its higher contrast and with earlier and stronger luminance OFF responses (24), again, conforming with an explanation of early evoked differences as the signature of contrast and adaptation to it, rather than of categorical processing. This time, our statistical analyses of ERP amplitudes in the vMMN window do reveal a three-way interaction in the linear mixed effect model: There is a significant difference between standards and deviants only for light “cool” colors (−0.762) µV, 95% CI [0.407,1.117]; t(519) = 4.210, *P* < 0.001; see *SI Appendix*, Supplementary Materials 3 for more details). This replicates our observation of higher N1 amplitudes for light blue and light green in the 1^st^ experiment. Thus, instead of the predicted vMMN enhancement for “warm” colors, we find the opposite pattern, although simulated power analysis (25) demonstrates we have only just enough power to observe this small effect (marginal R^2^ = 0.026, conditional R^2^ = 0.814; see *SI Appendix*, Supplementary Materials 3 for further details).

**Fig. 5.**
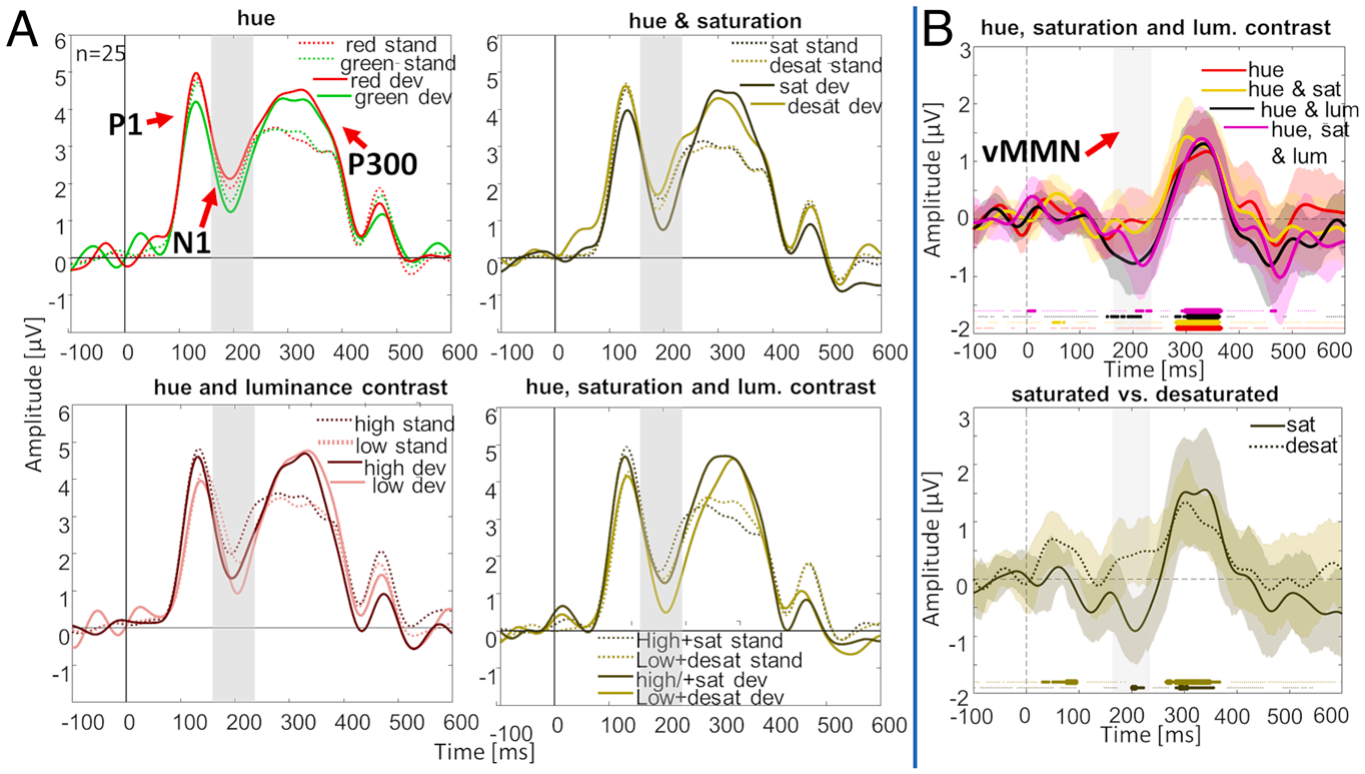
ERPs and difference waves from Experiment 3 examining the effects of hue, saturation, and lightness. As stimuli are defined in CIE LCh space, L (lightness) is equivalent to luminance contrast and C (chroma, or colourfulness) corresponds to saturation, with higher chromatic contrast for more saturated stimuli. See also *SI Appendix*, Fig. S1 for stimulus locations in both CIELAB and MacLeod–Boynton chromaticity diagram. (*A*) This panel depicts the grand-mean waveforms elicited by standards and deviants differing in only hue (red and green; *Top Left*), hue and saturation (*Top Right*), hue and lightness (*Bottom Left*) or all three dimensions (i.e., hue, saturation, and lightness; *Bottom Right*). The overall waveforms are similar to those in Figs. 2–4, with P1, N1, and P300 components. Note, however, the differences in P1 and N1 amplitudes and latencies, with P1 amplitude appearing to be higher and latency shorter for higher luminance contrasts. As expected, red also appears to elicit a higher P1 than green (26). (*B*) Difference waves, created by subtraction of deviants and standards in the four panels of Fig. 5*A* (reflecting differences in hue alone, or hue together with saturation and/or lightness) or separated between saturated and desaturated hues (*Bottom* panel). BF values are indicated above the x-axis, with smallest dots indicating lack of significant differences from zero (BF < 0.33), medium-sized dots indicating moderate evidence for a significant difference (3 < BF <10) and large dots indicating strong evidence of a deflection in the difference wave (BF > 10). Two points/aspects are of note: 1) The difference waves only exhibit a significant deflection in the N1 window when differences in luminance contrast are present. These differences are less robust across time for a combination of saturation and luminance contrast changes. 2) Averaging hues across saturation levels may conceal differences between saturated and desaturated colours. This could be the reason why combined effects of saturation and luminance contrast are less robust than for luminance contrast alone. The driver of these differences appears to be a more positive-going waveform for desaturated colours. In *SI Appendix*, Supplementary Materials 4, we show a summation analysis of chromatic and luminance contrast combinations. As can be expected, this summation is not linear (27). Note that the time-window of the N1 and vMMN components is highlighted with a gray overlay in all subplots.

Finally, to verify the observed effects of contrast on the vMMN, we conducted Experiment 3 in which we modulated hue, chromatic, and luminance contrast systematically. Consistent with the findings from our first two experiments, vMMN responses were primarily driven by contrast-based properties (Fig. 5). Bayesian hypothesis testing revealed no reliable effect of hue on the vMMN (BF_10_ = 0.197). Changes in saturation (chromatic contrast) elicited asymmetrical responses, with a more negative deflection observed only for saturated deviants relative to standards (Fig. 5*B*), while desaturated colors showed no such effect, yielding a nonsignificant overall vMMN for saturation (BF_10_ = 0.238). In comparison, for luminance contrast (darker colors had 30% contrast, as opposed to 84% contrast for lighter colors), we found strong evidence supporting the presence of the vMMN [BF(d < 0) = 10.059]. This, again, suggests that luminance contrast modulates vMMN amplitude, consistent with its dependence on contrast-adaptation mechanisms rather than on categorical color processing.

## Discussion

Our findings consistently and robustly demonstrate that color categories do not automatically influence early neural markers of color feature processing. In Experiment 1 we performed a close replication of Thierry et al.’s (5) widely cited study but with Russian rather than Greek participants, who also have two basic terms for light and dark “blue.” Although the two shades fall into separate “blue” categories for Russian participants (28), we fail to observe robust categorical modulations. In fact, through two further experiments that systematically examine the factors that modulate EEG activity in the time-window of the early, perception-related processing of color, we observe a series of consistent modulations by adaptation to luminance and color contrast, with no modulations by basic color categories. Our findings do not only challenge psychological models that posit an early, preattentive automatic influence of cognition on perception (11) but also raise questions that concern models of predictive coding (9), which propose some of the same visual contrast-driven EEG components as neural correlates of prediction.

How can we reinterpret the original finding of Thierry et al. (5) in light of our failed replication? Their study did not fully consider that the vMMN could be driven by factors other than categorical stimulus properties, e.g., lightness of a color. If lightness was included in their statistical models, the authors would have observed that it appears to be the critical factor in eliciting the vMMN. The effects of lightness are not consistent with categorical effects and imply a more basic explanation for drivers of EEG differences. Our Experiment 3, manipulating hue, chromatic, or luminance contrast, demonstrates that the vMMN is not robustly present for categorically distinct colors in red and green areas of color space, which represent a highly salient categorical difference, but, rather, depends on chromatic and luminance contrast. Notably, while stimuli differing in luminance contrast elicit a robust vMMN, irrespective of whether the deviant or the standard has higher luminance contrast, for chromatic contrast the vMMN is elicited only for the higher-contrast deviant among lower-contrast standards. We know from normative studies that chromatic contrast is associated with negative deflections in the early ERP and lacks an initial positive peak (29, 30). The asymmetric effect of refractory activity following adaptation that we report here, to our knowledge, has not been previously reported. Asymmetric interactions between chromatic and luminance contrast are well known (31–33) and their manifestations in early electrophysiological markers of adaptation warrant further investigation.

If color categories are not activated automatically, then the key determinant for their activation is likely to be the task that the participant is performing. It has been argued that this should be a task that involves language-derived color concepts (14). This does pose challenges when probing categorical color representation in preverbal infants or nonhuman animals, with recent studies opting for tasks reliant on recognition memory in preverbal infants (34) or working memory in macaques (35), since categorical biases are amplified when maintaining information in visual working memory (36). Future electrophysiology studies of color categories should follow the Hillyard principle (7): Using the same stimulus, they should examine the difference between perceptual tasks and tasks that require comparisons of a presented color either to a previously seen sample, or to an internal, language-derived standard.

High susceptibility of the vMMN to contrast-adaptation effects is also very relevant for models of predictive coding. These models take MMN to be the signature of prediction across all sensory modalities (9). Male et al. (16) argue that the positive difference at ~100 ms may be the signature of prediction for basic featural changes, instead of the vMMN. However, we demonstrate that even this positive deflection is contrast-related, since it is observed reliably only when stimuli vary in contrast and luminance polarity, and that its modulations are not independent of the subsequent modulations in the N1 window (*SI Appendix*, Fig. S4). In fact, one could even argue that the oddball task, as employed by Thierry and colleagues (5), cannot elicit a genuine vMMN since the stimuli are all in the focus of attention. This would be corroborated by large P300 deflections that appear enhanced for deviants as opposed to standards (Figs. 2–5). Future studies will need to perform systematic tests of contrast vs. feature responsiveness at different retinal locations to establish whether foveal vMMN exists independently of contrast (37, 38).

In conclusion, our results reliably, consistently and systematically confirm the crucial role of contrast and contrast adaptation, rather than categorical basicness, in driving early event-related activity in the EEG in the first 200 ms of visual processing. This refutes some of the strongest evidence in favor of the models that propose a high degree of cognitive penetrability of perception (5, 11). Indeed, further studies of cognitive penetrability of perception could benefit from testing their predictions in the color vision domain, where much is already known about the low-level drivers of visual evoked potentials (39), hence, potential low-level confounds are easier to predict and account for. This also applies to the search for neural markers of predictive coding. In fact, it would be highly interesting if in the visual domain they are built upon the probabilistic read-out of other more basic and general biological mechanisms such as contrast adaptation (40). Having contrast adaptation at the root of prediction is not inconsistent with computational models of adaptation that involve “unaware” read-outs (41). Indeed, examining the contributions of sensory adaptation to prediction anomalies would be a highly fruitful avenue for future research, of particular relevance for clinical applications of the model, which presume aberrant predictive coding in psychosis and often rely on MMN measures (42, 43).

## Methods

### Participants.

In Experiment 1, 27 native Russian participants recruited from a university student population (10 male; all right-handed; mean age 23, range 19 to 25) completed Experiment 1, which was a close replication of Thierry et al. (5). Three further participants were removed from the sample: One did not complete the experiment, another had an excessive target miss rate (56%), and still other produced insufficient artifact-free trials. All participants had normal or corrected-to-normal vision and showed normal color vision assessed by the Farnsworth-Munsell 100 Hue test. Before participating, they gave written informed consent. The experiment was approved by the Ethics Committee of Smolensk State University.

Experiments 2 and 3 were conducted at the University of Edinburgh, with study protocols approved by the Psychology Ethics Committee. In the conceptual replication with English speakers (Experiment 2), 36 participants (6 male, 1 nonbinary; 4 left-handed; mean age 25, range 19 to 36) were kept in the final sample, with 5 participants removed due to technical issues during the recording. Our final, preregistered follow-up experiment on color feature determinants of the vMMN (Experiment 3; https://osf.io/ymk9u/) included 25 participants (9 male, 2 left-handed, mean age 26, range 18 to 59) in the final sample, with 1 further participant removed due to an incomplete EEG session. All participants were recruited through posters around the university campus and word-of-mouth. They reported normal or corrected-to-normal visual acuity and had adequate color vision, as assessed with the diagnostic plates from the City University test (44). Participants gave written informed consent and were reimbursed for their participation.

All three studies were conducted in line with the Declaration of Helsinki (1964) (45).

### Apparatus, Stimuli, and Procedure.

Experimental stimuli and procedures were aligned as much as possible between the two experimental sites (Smolensk and Edinburgh), despite the reliance on different types of apparatus and EEG recording systems.

Experiment 1 (Smolensk) was run on an X-Rite™ Pantone® certified factory calibrated AERO 15 OLED 15.6-inch monitor with a screen resolution of 3,840 × 2,160 pixels at a refresh rate of 144 Hz viewed from a distance of 70 cm in a dark room with no other light source. Accuracy of color reproduction on the monitor during the experiment was controlled with the help of The Calibrite ColorChecker Display Pro device and software. The experiment was run using Psychophysics Toolbox Version 3 (PTB-3) software that extends Matlab (Mathworks) with functions for research-grade stimulus presentation and response collection (46, 47). A VOROTEX K03 Red Switch (Vorotex, China) response pad with a simplified 3-key layout was used to collect behavioral data.

Experiments 2 and 3 (Edinburgh) were conducted using a calibrated Display++ screen [Cambridge Research Systems (CRS), UK] controlled by a ViSaGe visual stimulus generator (CRS, UK). The experiments were run using the CRS toolbox and CRS color toolbox (48) for Matlab (Mathworks). Measurements of monitor spectra were obtained using a SpectroCAL (CRS, UK) spectroradiometer. Participants were seated at a 70-cm distance from the display, which was positioned in a dark room with no other light source. They provided responses using a Cedrus-RB530 button box (Cedrus).

Stimulus colors are depicted in Fig. 1. Stimuli subtended 2° visual angle enabling use of 2° color matching functions for calculating color coordinates via the Optprop toolbox for Matlab (49). As in Thierry et al. (5), a gray background was set to CIE 1931 coordinates 0.3128, 0.3290, 26.2 cd/m^2^.

In Experiment 1, colors were identical to those used by Thierry et al. (CIE 1931, dark blue: 0.234, 0.230, 10.7 cd/m^2^, light blue: 0.259, 0.264, 41.5 cd/m^2^, dark green: 0.259, 0.397, 10.7 cd/m^2^, light green: 0.279, 0.377, 41.7 cd/m^2^). Luminance contrast against the background amounted to 59%, with LCh transformation of CIELAB coordinates for lightness (L), chroma (C), and hue (h) equal to L = 39 for dark colors, L = 71 for light colors, and C = 28, h = 281° for dark blue, C = 28, h = 278° for light blue, C = 29, h = 166° for dark green, and C = 31, h = 164° for light green. Transformation into CIELUV-derived LCh reveal a higher discrepancy between chroma of light and dark blues (46 vs. 41) and greens (39 vs. 31). The differences in chroma and hue between Munsell colors of different lightness, when transformed into CIELAB and CIELUV, are outcomes of the underlying transformations. Among other things, these calculations expand colorfulness at higher luminance levels (50). While we did not ask the participants in the EEG experiment to free-name the color samples at the end of the experiment, we recruited a separate sample of 76 Russian speakers for this task. The dark blue stimulus was named sinij or tëmno-sinij “dark sinij” by 81% participants, while the light blue stimulus was labeled goluboj by 32% of observers, with another 42% using different compounds containing goluboj or hyponyms of the goluboj category (for full naming data, see *SI Appendix*, Supplementary Materials 2). These color naming patterns are in line with previous work with Russian speakers (51).

In Experiment 2, color coordinates were defined in CIELAB with these two aims in mind: 1) to ensure approximately equal saturation across hues by setting all colors to have equal chroma (C = 27) and hue angles (green h = 155°, blue h = 254°, red h = 0°, and yellow h = 101°); 2) to introduce a small difference in luminance contrast (with luminance of 10.08 cd/m^2^ for dark and 39.34 cd/m^2^ for light colors, equal to 62% and 50% contrast against the background, respectively): While insufficient to alter the relational (light or dark) or categorical status (yellow or brown), such luminance contrast should still be sufficient to affect the EEG response systematically due to varying response properties for luminance ON and luminance OFF neurons (24). CIELAB was chosen as it accounts for differences in saturation more effectively than some other color spaces (52).

We verified that colors indeed belonged to the same or different categories by asking participants to free-name all color samples at the end of the experiment. Color circles were presented on the screen in a randomized order; we report frequency and percentage of basic color terms assigned to them, as well as of hyponyms, modified, compounded, or “fancy” terms that could be allocated to those same categories (e.g., periwinkle for light blue; see *SI Appendix*, Supplementary Materials 3 for the full list of names). Light and dark blue stimuli were named blue by 21 (58%) and 29 (81%) participants, respectively, with the remaining participants generally using nonbasic terms that would fall under light or dark blue. A similar picture emerged for light and dark green, which were named green by 16 (44%) and 28 (78%) participants, respectively. Pink was offered by 27 (75%) of observers, while in naming of the red sample both pink (10 participants, 28%) and red (8 participants, 22%) were prominent [for similar patterns of pink-naming, see Rosenthal et al. (53)]. Yellow was elicited in 30 (79%) participants, while naming of the brown stimulus was split across two basic terms, with 11 participants (31%) opting for green and the same number opting for brown. An increase of greenness in yellow colors with reduction of luminance was to be expected (54).

In Experiment 3, we selected our colors so as to manipulate hue, saturation, and luminance contrast selectively—either in isolation or in combination with each other—to test the effects observed in the first two experiments in a principled manner. This experiment was preregistered prior to data collection, meaning that the hypothesis, design, and analysis plan were publicly specified on the Open Science Framework (OSF; https://osf.io/ymk9u/). Colors—red and green—were defined in CIELAB LCh coordinates, which allowed us to systematically vary perceptual properties across blocks. Red was always set to h = 0° and green to h = 180° in CIE LCh. The experiment consisted of 16 blocks, organized into 8 block pairs, with standard and deviant roles reversed within each pair between red and green. Blocks 1 to 2 and 3 to 4 had stimuli of fixed lightness and chroma (L = 75, C = 27 for both red and green). In blocks 5 to 6 and 7 to 8, both hue and chroma were manipulated: In blocks 5 to 6, green was desaturated (L = 75, C = 27 for red and L = 75, C = 10 for green), while in blocks 7 to 8 red was desaturated (L = 75, C = 10 for red and L = 75, C = 27 for green). Blocks 9 to 10 and 11 to 12 manipulated hue in combination with luminance contrast, lowering lightness while keeping chroma constant (L = 75, C = 27, h = 0° vs. L = 65, C = 27, h = 180°; and L = 65, C = 27, h = 0° vs. L = 75, C = 27, h = 180° respectively). The luminance contrast manipulation amounted to 30% contrast for the darker colors, as opposed to 84% contrast for the lighter colors. Finally, blocks 13 to 14 and 15 to 16 introduced combined changes in all three dimensions—hue, chroma, and lightness. In blocks 13 to 14, both chroma and lightness were reduced for one hue (L = 75, C = 27, h = 0° vs. L = 65, C = 10, h = 180°), and in blocks 15 to 16, the asymmetry was reversed (L = 65, C = 10, h = 0° vs. L = 75, C = 27, h = 180°). Each block comprised 225 trials, of which 45 were deviant (either by color, or shape, or combination of both).

Our experimental procedure followed closely the template provided by Thierry et al. (5). Stimuli were presented for 200 ms with an interstimulus interval of 800 ms. Streams of 3, 4, or 5 standard stimuli were followed by a randomly selected oddball stimulus resulting in stimulus streams with 6.67% oddballs of each type (shape only oddball, luminance, and shape oddball, luminance only oddball). Participants were instructed to respond to shape oddballs (i.e., squares) and ignore circles. The stream contained the following total numbers of stimuli: 2,700 in four blocks of 675 trials, which included 540 standards and 135 deviants (45 of each type) in Experiment 1; 3,600 in eight blocks in Experiment 2, with 450 standards and 90 deviants of each color (30 of each type; thus 60 nontarget deviants, when collapsed across “warm” and “cool” hues) in Experiment 2; and 3,600 in Experiment 3, separated into 16 blocks. Block order was randomized across participants. One participant from Experiment 1 was excluded due to low hit rate (<75%). Participants remaining in the sample performed the task with a high degree of accuracy (Experiment 1: 86.0% correct rejections, 0.7% false alarms, 13.0% hits, 0.3% misses, reported as a percentage out of a total of 100%, with average reaction time (RT) and SD 600 ± 115 ms; Experiment 2: 86.5% correct rejections, 0.2% false alarms, 12.9% hits, 0.4% misses, RT 423 ± 95 ms; Experiment 3: 86.49% correct rejections, 0.18% false alarms, 13.06% of hits and 0.27% misses, RT 444 ± 98 ms).

### EEG Recording and Preprocessing.

In Smolensk (Experiment 1), electrophysiological data were recorded continuously using an eego mylab 64-channel amplifier and waveguard original EEG caps, with 64 Ag/AgCl electrodes placed according to the extended 10 to 20 system. Data were sampled at 250 Hz. Bipolar horizontal (HEOG) and vertical (VEOG) electrooculograms were recorded simultaneously to monitor eye movements. Impedance of all electrodes was kept below 10 kΩ. Stim Tracker (Cedrus, San Pedro, CA) was used to synchronize stimuli with the EEG data, bypass delays associated with operating systems and accurately record true onset time of the stimuli. In Edinburgh (Experiments 2 and 3), continuous EEG was recorded using a 64-channel BioSemi Active-Two (Biosemi, Netherlands) amplifier system, digitizing the signal at a 1,024 Hz sampling rate. 64 Ag-AgCl electrodes were mounted in an elastic, tight-fitting cap. In addition, we also recorded HEOGs and VEOGs, and two mastoid references. Stimulus triggers were sent from the Display++ device, synchronized to stimulus onset using Visage Framestore cycling functions.

Data from both sites were analyzed in Matlab (Mathworks) using the EEGLAB toolbox (55). FASTER (56) and ADJUST (57) toolboxes were used for artifact rejection and correction. Initially the data were average-referenced and filtered using a 40 Hz low-pass and 0.1 Hz high-pass Hamming windowed sinc Finite Impulse Response (FIR) filters, as implemented in EEGlab (55). Recordings were then separated into epochs representing individual trials, 1,000 ms in duration. This consisted of a 200-ms period before the stimulus onset and 800 ms thereafter. We also applied a baseline correction between −100 ms and 0 ms. Contaminated trials were identified and rejected using the FASTER toolbox. This is done by characterizing global channel properties using statistical parameters of the data and applying a metric of ±3 z-score as a marker for contaminated data. Independent component analysis (ICA) was then performed using the default runica algorithm. The ADJUST toolbox was used to identify specific components driven by blinks, eye movements, and local discontinuities. These were removed from the data. Following artifact rejection, FASTER was used to identify any contaminated channels and interpolate them from neighboring channels. A threshold of ±3 was applied to the F-score parameter to define these outliers, which were removed and interpolated. A manual check was then performed on the data to verify quality of the artifact rejection procedure. In Experiment 1, percentage of rejected trials ranged between 1% and 5%; in Experiment 2, this was between 1% and 7%, and in Experiment 3 between 1% and 5%. As in Thierry et al. (5), the vMMN was quantified over parieto-occipital electrodes: In Experiment 1, these were O1, O2, Oz, PO7, and PO8, while in Experiments 2 and 3, Iz was also available in the electrode montage and, thus, added to this electrode cluster. Time-windows for statistical analyses were selected to encompass the N1 peak from the grand-mean ERP plots: 188 to 220 ms in Experiment 1, 172 to 223 ms in Experiment 2, and 164 to 234 ms in Experiment 3. The slight differences in the time-windows stem from the need to encompass the N1 component of the ERP, which varied in latency along expected lines in those experiments that manipulated contrast (30, 58).

### Statistical Data analysis.

We fitted linear mixed effects models (LMEMs) to our data, with random by-participant intercepts to model overall differences in amplitude between observers and fixed effects of deviancy, hue, and lightness (in Experiment 3, there was an additional fixed effect of saturation) to model our experimental manipulations, with simple coding of effects. To evaluate whether the ERP difference wave manifested a reliable difference from zero, we used Bayesian *t* tests (47). Other statistical analyses were implemented in R 4.3.1 (59) using the following packages: lmerTest 3.1-3 (60), emmeans 1.10 (61), DHARMa 0.4.6 (62), simr 1.0.7 (63), and BayesFactor 0.9.12 (64). For further details of statistical analysis, see *SI Appendix*, Supplementary Materials 2–4, where we also report statistical sensitivity/power analyses and all the properties of best fitting models.

## Supplementary Material

Appendix 01 (PDF)

## Data Availability

Grand mean EEG and behavioural data have been deposited in OSF (65).
