# Supplementary material for: Cognition does not automatically influence perception: Evidence from neural encoding of colors belonging to different categories
Source: Proc Natl Acad Sci U S A. 2026 Jun 9;123(24):e2538139123. doi: 10.1073/pnas.2538139123 (PMC13273331; doi:10.1073/pnas.2538139123)
Supplement: Supplementary file 1 — Appendix 01 (PDF) [file pnas.2538139123.sapp.pdf]

Supplementary Materials

Cognition does not automatically influence perception:

Evidence from neural encoding of colours belonging to different categories

by Martinovic et al.

Supplementary Material 1 – Stimulus locations in the CIELAB space and Macleod-Boynton chromaticity diagram across all three experiments

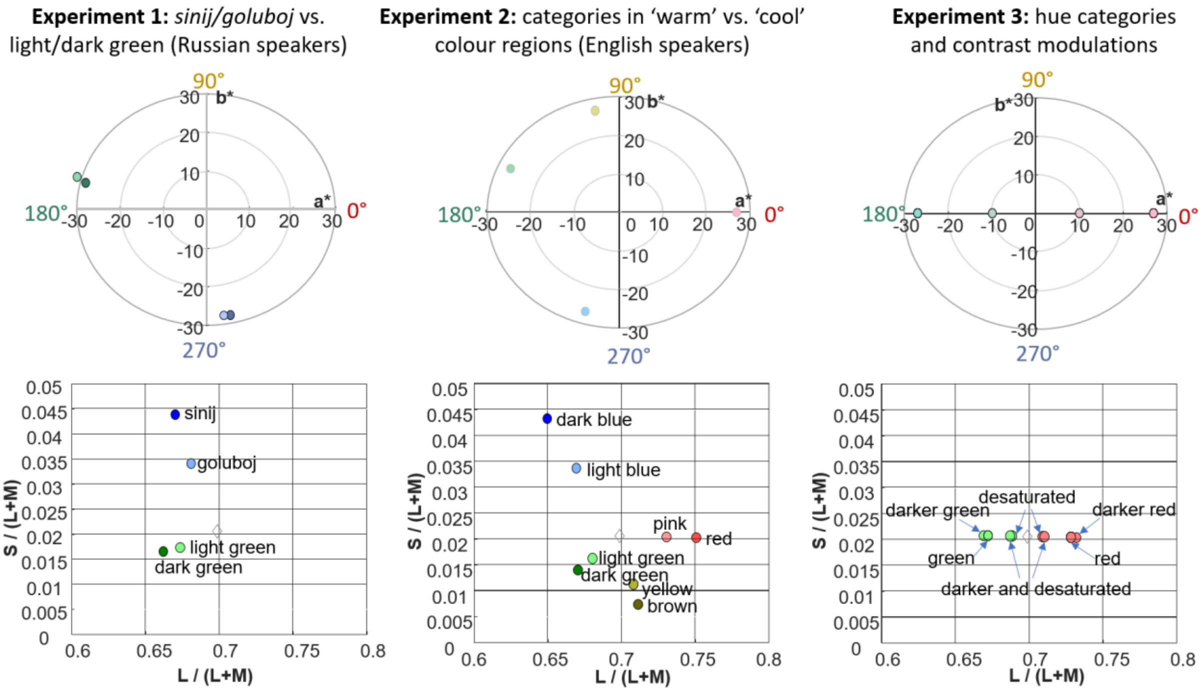

*Figure S1. Top panel: Colour coordinates in the a*b* colour plane of the CIELAB space. For a full depiction of the CIELAB space, see Figure 1. a* captures the red (0°) – green (180°) dimension, while b* captures the blue (270°) - yellow (90°) dimension of hue. Distance from the centre of the space is equivalent to chroma. Bottom panel: Colour positions in the MacLeod-Boynton (MB) chromaticity diagram, whose axes depict L-cone (x) and S-cone (y) excitations, relative to the sum of the L and M-cone excitations. Note that green and red colours in Experiment 3 occupy approximately opposite locations relative to the background on the L axis. In CIELAB, our background's chromaticity coincides with the centre of the space and in the MB diagram it is denoted with the grey diamond.*

Supplementary Material 2 – Effect of additional basic colour categories in the blue area of colour space (Experiment 1)

Best fitting linear mixed effect model: Fixed effects of deviancy (standard – reference level, or deviant) and lightness (dark – reference level, or light) and random by-participant intercepts only.

Model: amplitude ~ deviancy*lightness + 1|participant

	**Amplitude (μV)**		
Predictors	Estimates	95% CI	P
(Intercept)	-0.12	[(-0.86), 0.61]	0.742
Deviancy	-0.49	[(-0.75),(-0.23)]	**<0.001**
Lightness	-0.81	[(-1.07),(-0.56)]	**<0.001**
Deviancy × Lightness	-0.95	((-1.46),(-0.43)]	**0.001**
Random Effects			
σ^2	0.87		
$\tau_{00\ participant}$	3.51		
ICC	0.80		
$N_{participant}$	26		
Observations	205		
Marginal R^2 / Conditional R^2	0.061 / 0.814		

Prior to statistical analyses, we rejected any datapoints that were outliers: this resulted in rejection of 11 datapoints, which included all the datapoints from one participant (as can be seen from N and Observation rows in the table above).

Assumption checks were performed using the DHARMa R package and confirmed that the data were appropriate for the fitted model. We also verified that the predictors were not correlated by fitting an additive model and evaluating it with the *check_collinearity* function from the performance R package.

Initially, we fitted a model with the full set of fixed effects (deviancy*colour*lightness), but the step function from lmerTest R package indicated that colour (with two levels: blue and green) did not contribute significantly to the model, individually or in interaction with the other two factors:

Deviancy:Colour:Lightness, F(1, 171.98) = 3.4135, p = 0.066

Deviancy:Colour, F(1, 172.98) = 0.0339, p = 0.854

Colour:Lightness, F(1, 173.98) = 0.631, p = 0.428

Colour, F(1, 174.98) = 0.0436, p = 0.835

On the other hand, deviancy and lightness interacted significantly and therefore could not be removed from the model (F(1,175.98)=12.963, p<.001).

Power analysis: Sensitivity analyses were performed by resampling the number of participants (1000 simulations) and evaluating the observed power for obtaining the statistically significant fixed effects in the best fitting model, using the R package *simr*. Sufficient power is reached within 16 participants for the interaction of deviancy and lightness (see Figure S2, plotting simulated power on the y- axis against number of participants on the x-axis). We also evaluated the power to detect the observed effects if they were reduced by 15%, as advised by Kumle and colleagues(25)), and found that this was 91.70% (95% CI [89.81%,93.34%].

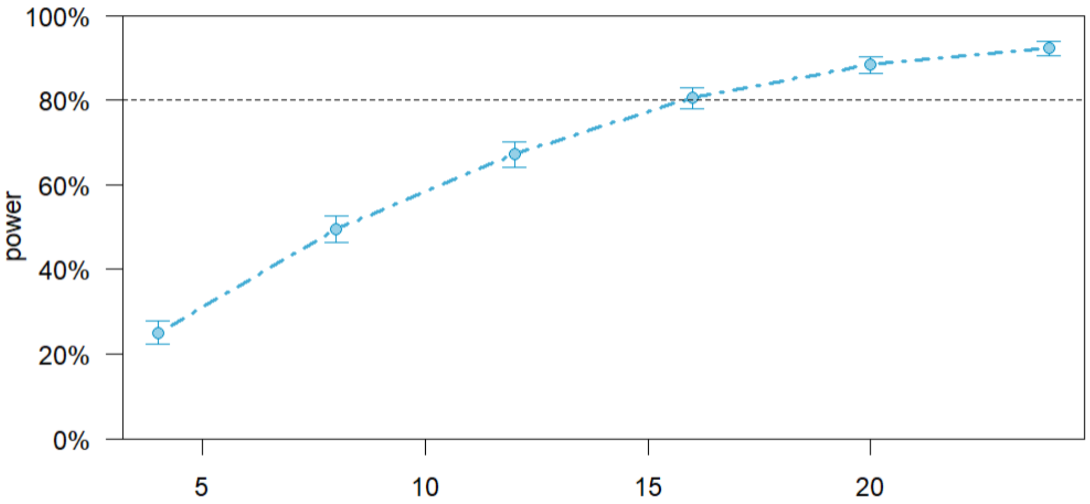

Figure S2. Power analysis for Experiment 1: deviancy by lightness interaction.

Free colour naming of colour circles by Russian participants:

We verified that colours indeed belonged to the same or different ‘blue’ categories by asking 76 native Russian speakers (25 male; all right-handed, mean age 22, range 19-25) to free-name the four colour samples in a separate naming experiment, approved by the Ethics Committee of Smolensk State University. Colour circles were presented on the screen in a randomised order.

In Tables S1-4 show frequency (F) and relative frequency (%F) of colour terms assigned to each stimulus. Russian colour names were transliterated into Latin script using the free Online Transliterator (https://translit.cc). English glosses followed Frumkina and Mikhejev (1996).

We obtained the largest numbers of different colour names (basic colour terms, as well as hyponyms, modified, compounded, and ‘fancy’ terms) for the light blue (N=21) and light green (N=17) stimuli. The sets of colour names obtained for dark stimuli were more consistent and included 14 terms for dark blue and 13 for dark green.

Light green stimulus was named *svetlo-zelënyj* ‘light green’ or *zelënyj* ‘green’ by 61% participants, with the remaining participants generally using non-basic terms and compounds that would fall under light green (*mâtnyj* ‘mint’, *salatovyj* ‘lettuce-coloured’, *zelëno-birûzovyj* ‘green turquoise’, *bledno-birûzovyj* ‘pale turquoise’, *bledno-izumrudnyj* ‘pale emerald’, *bledno-zelënyj* ‘pale green’, *cvet morskoj volny* ‘colour of sea wave’, *izumrudnyj* ‘emerald’, *malaxitovyj* ‘malachite’, *svetlo-birûzovyj* ‘light turquoise’, *svetlo-izumrudnyj* ‘light emerald’, *svetlo-malaxitovyj* ‘light malachite’, *svetlo-salatovyj* ‘light-lettuce-coloured’, *tëmno-mâtnyj* ‘dark mint’, *xaki* ‘khaki’) (Table S1).

Table S1. Full list (N=17) of Russian colour terms elicited for the light green stimulus with English glosses. Each term is provided with its absolute frequency (F) and relative frequency (%F), in descending order. Colour descriptors containing *zelënyj* ‘green’ are shaded.

**Colour name**	**English gloss**	**F**	**%F**
svetlo-zelënyj	light green	24	32%

zelënyj	green	22	29%
mâtnyj	mint	13	17%
salatovyj	lettuce-coloured	2	3%
zelëno-birûzovyj	turquoise green	2	3%
bledno-birûzovyj	pale turquoise	1	1%
bledno-izumrudnyj	pale emerald	1	1%
bledno-zelënyj	pale green	1	1%
cvet morskoj volny	colour of sea wave	1	1%
izumrudnyj	emerald	1	1%
malaxitovyj	malachite	1	1%
svetlo-birûzovyj	light turquoise	1	1%
svetlo-izumrudnyj	light emerald	2	3%
svetlo-malaxitovyj	light malachite	1	1%
svetlo-salatovyj	light-lettuce-coloured	1	1%
tëmno-mâtnyj	dark mint	1	1%
xaki	khaki	1	1%

A similar picture emerged for dark green, which was named *tëmno-zelënyj* ‘dark green’ or *zelënyj* ‘green’ by 71% participants, while another 5% of participants used the term *zelënyj* ‘green’ as part of compounds *bolotno-zelënyj* ‘marsh green’, *izumrudno-zelënyj* ‘emerald green’, *malaxitovo-zelënyj* ‘malachite green’, *nasyŝennyj tëmno-zelënyj* ‘saturated dark green’, *zelënyj kak trava* ‘grass green’ (Table S2).

Table S2. Full list (N=13) of Russian colour terms elicited for the dark green stimulus with English glosses. Each term is provided with its absolute frequency (F) and relative frequency (%F), in descending order. Colour descriptors containing *zelënyj* ‘green’ are shaded.

**Colour name**	**English gloss**	**F**	**%F**
tëmno-zelënyj	dark green	39	51%
zelënyj	green	15	20%
izumrudnyj	emerald	9	12%
bolotnyj	marsh	3	4%
tëmno-izumrudnyj	dark emerald	2	3%

bolotno-travânoj	marsh grass green	1	1%
bolotno-zelënyj	marsh green	1	1%
izumrudno-zelënyj	emerald green	1	1%
kamuflâžnyj	camouflage	1	1%
malaxitovo-zelënyj	malachite green	1	1%
nasyŝennyj tëmno-zelënyj	saturated dark green	1	1%
xvojnyj	conifer	1	1%
zelënyj kak trava	grass green	1	1%

The light blue stimulus was labelled *goluboj* by 32% of observers, while other 42% used different compounds containing *goluboj*: *svetlo-goluboj* ‘light *goluboj*’ (12%), *nebesno-goluboj* ‘sky *goluboj*’ (12%), *pastel'no-goluboj* ‘pastel *goluboj’* (7%), *sero-goluboj* ‘grey-*goluboj’* (3%), *blednyj nebesno-goluboj,* ‘pale sky *goluboj’*, *bleklo-goluboj* ‘faded *goluboj’*, *cvet golubyx džins* ‘colour of *goluboj* jeans’, *golubo-lilovyj* ‘*goluboj*-mauve’, *golubo-seryj* ‘*goluboj*-grey’, *golubovato-sirenevyj* ‘*goluboj*-ish-lilac’, *sirenevo-goluboj* ‘lilac-*goluboj’*, *tëmno-goluboj* ‘dark *goluboj’* (1% each) (Table S3).

Table S3. Full list (N=21) of Russian colour terms elicited for the light blue stimulus with English glosses. Each term is provided with its absolute frequency (F) and relative frequency (%F), in descending order. In compound and modified terms containing *goluboj* and *sinij*, the original Russian form is retained in the gloss to indicate their denotative distinction. Colour descriptors containing *goluboj* are shaded.

**Colour name**	**English gloss**	**F**	**%F**
goluboj	*goluboj*	24	32%
svetlo-goluboj	light *goluboj*	9	12%
nebesno-goluboj	sky *goluboj*	9	12%
nebesnyj	sky-coloured	8	11%
pastel'no-goluboj	pastel *goluboj*	5	7%
svetlo-sinij	light *sinij*	3	4%
sero-goluboj	grey-*goluboj*	2	3%
lazurnyj	azure	2	3%
vasil'kovyj	cornflower blue	2	3%
blednyj nebesno-goluboj	pale sky *goluboj*	1	1%
bleklo-goluboj	faded *goluboj*	1	1%

cvet golubyx džins	colour of *goluboj* jeans	1	1%
golubo-lilovyj	*goluboj*-mauve	1	1%
golubo-seryj	*goluboj*-grey	1	1%
golubovato-sirenevyj	*goluboj*-ish-lilac	1	1%
lavandovyj	lavender	1	1%
ledânoj	Icy	1	1%
nalët so slivy	plum bloom	1	1%
sirenevo-goluboj	lilac-*goluboj*	1	1%
svetlyj džinsovyj	light denim	1	1%
tëmno-goluboj	dark *goluboj*	1	1%

Dark blue stimulus was named as *sinij* or *tëmno-sinij* ‘dark *sinij’* by 81% participants, with other 12% opting for *sinij* as a part of compounds: *bledno-sinij* ‘pale *sinij*’, *kosmičeskij sinij* ‘space *sinij’* (3% each), *korolevskij sinij* ‘royal *sinij*’, *priglušennyj tëmno-sinij* ‘muted dark *sinij*’, *prusskij sinij* ‘Prussian *sinij*’, *pyl'no-sinij* ‘dusty *sinij*’, *sero-sinij* ‘grey-*sinij*’, *sine-seryj* ‘*sinij*-grey’ (1% each) (Table S4).

Table S4. Full list (N=14) of Russian colour terms elicited for the dark blue stimulus with English glosses. Each term is provided with its absolute frequency (F) and relative frequency (%F), in descending order. In compound and modified terms containing *sinij*, the original Russian form is retained in the gloss to indicate its denotative distinction from *goluboj*. Colour descriptors containing *sinij* are shaded.

**Colour name**	**English gloss**	**F**	**%F**
tëmno-sinij	dark *sinij*	33	43%
sinij	*sinij*	29	38%
bledno-sinij	pale *sinij*	2	3%
kosmičeskij sinij	space *sinij*	2	3%
černičnyj	blueberry coloured	1	1%
kobal'tovyj	cobalt	1	1%
korolevskij sinij	royal *sinij*	1	1%
nočnoe nebo	night sky	1	1%
priglušennyj tëmno-sinij	muted dark *sinij*	1	1%
prusskij sinij	Prussian *sinij*	1	1%

pyl'no-sinij	dusty *sinij*	1	1%
sero-sinij	grey-*sinij*	1	1%
sine-seryj	*sinij*-grey	1	1%
tëmno-fioletovyj	dark purple	1	1%

Supplementary Material 3 – Differences between 'warm' and 'cool' colours, which differ in basic colour terms across light and dark examples (Experiment 2)

Best fitting linear mixed effect model: Fixed effects of deviancy (standard – reference level, or deviant), colour (cool – reference level, or warm) and lightness (dark – reference level, or light) and random by-participant intercepts only.

Model: amplitude ~ deviancy*lightness*colour + 1|participant

	Amplitude (μV)		
Predictors	Estimates	95% CI	P
(Intercept)	1.56	[0.83,2.28]	**<0.001**
Deviancy	-0.20	[(-0.38),(-0.02)]	**0.029**
Colour	-0.04	[(-0.21),0.14]	0.689
Lightness	-0.50	[(-0.68),(-0.32)}	**<0.001**
Deviancy × Colour	0.11	[(-0.24),0.47]	0.525
Deviancy × Lightness	-0.24	[(-0.59),0.12]	0.193
Colour × Lightness	0.87	[0.51, 1.22]	**<0.001**
Deviancy × Colour × Lightness	1.56	[0.85,2.27]	**<0.001**
Random Effects			
σ^2	1.14		
$\tau_{00\ participant}$	4.86		
ICC	0.81		
$N_{participant}$	36		
Observations	562		
Marginal R^2 / Conditional R^2	0.026 / 0.814		

Prior to statistical analyses, we rejected any datapoints that were outliers: this resulted in rejection of 14 datapoints (as can be seen from Observations row in the table above).

Assumption checks were performed using the DHARMa R package and these confirmed that the data were appropriate for the fitted model. We also verified that the predictors were not correlated by fitting an additive model and evaluating it with the *check_collinearity* function from the performance R package.

The best fitting model is identical to the full model we initially fitted, as there was a significant 3-way interaction between colour, lightness and deviancy ($F(1,518.58)=18.506$, $p<.001$). Figure S3 depicts this interaction, with Bayesian stats pictorially coded by the dot size (smallest dot – evidence of absence, BF<0.33; medium dot –moderate evidence for a significant difference (3 < BF < 10); large dot – strong evidence of a deflection in the difference wave, BF > 10):

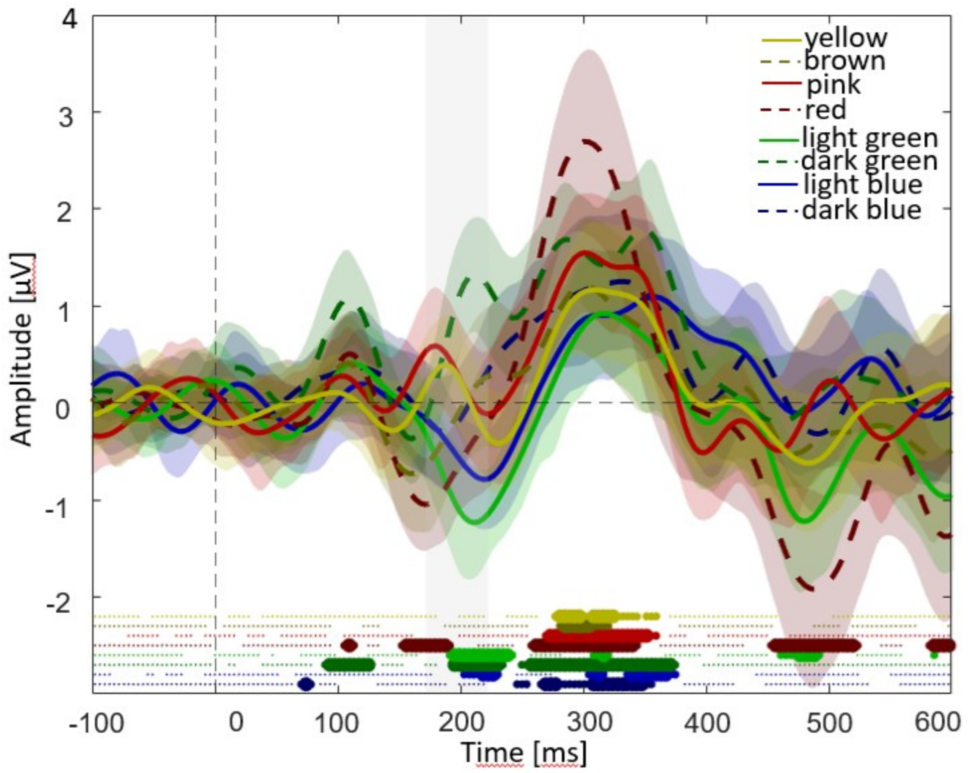

Figure S3. Difference waves elicited by dark and light versions of each colour, with the vMMN window indicated by a shaded grey area around 200 ms. Colour-shaded areas around each wave indicate the 95% between-subject CIs. As in the plots in the main manuscript, Bayes factor values are indicated above the x axis.

The plot reveals that the three-way interaction between colour, lightness and deviancy is likely to be driven by larger negative deflections for lighter and cooler colours. The difference wave for red also appears to be significantly more negative, but in a slightly earlier time-window, while the brown, yellow and pink difference waves did not exhibit an early negativity.

Finally, we also analysed if the difference waves depended on the order of the standard in the stimulus train – as the non-target deviant always followed a stream of 3, 4 or 5 standards. This is depicted in Figure S4 below.

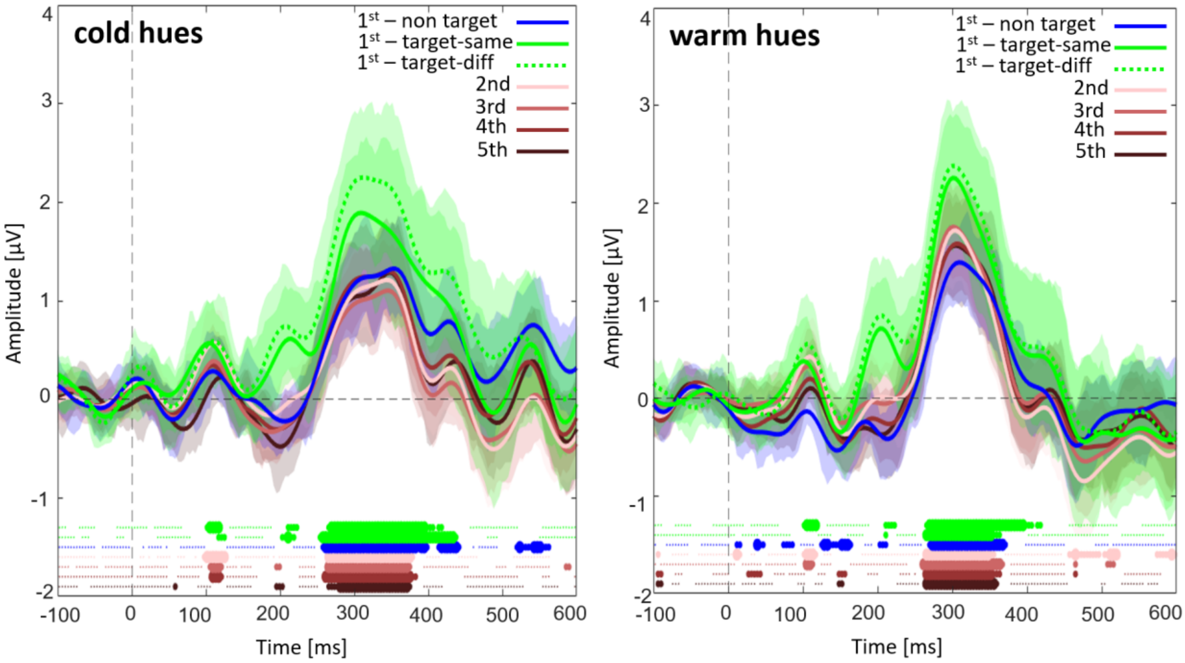

Figure S4. Difference waves for cool (left) and warm (right) hues calculated separately for each standard (1st-5th, with initial standards also separated depending on the preceding deviant, so that 1st -non-target is the 1st standard preceded by a non-target deviant, 1st target-same is the first standard preceded by a same colour target, and 1st target-different is the 1st standard preceded by a different colour target). For both types of hues, responses significantly different to baseline in the P1 window are elicited by 1st standards that follow target stimuli (i.e., squares) and 2nd standards – in parallel, strongest negative responses in the N1 window are observed for the 5th standards. Furthermore, 1st standards that follow a target elicit a positive peak in the difference wave in the same time-window as the vMMN. This is likely the reason why trials following a target are often excluded from vMMN analysis (e.g., Male et al., 2020).

Power analysis: Sensitivity analyses on the best fitting model were performed by resampling the number of participants (1000 simulations) and evaluating the observed power for obtaining the statistically significant three-way interaction, using the R package *simr*. We evaluated the power to detect the observed 3-way interaction if effect sizes were reduced by 15%, as advised by Kumle and colleagues (2021), and found that this was 68.50% (95% CI [65.52%, 71.37%]); for effect sizes reduced by 10%, we had 76.10% power (95% CI [73.33, 78.71%]) and for effect size reduced by 5% we had 79.20% power (95% CI [76.55, 81.68%]).

Power simulations therefore show that we have just about enough power to observe a three-way within-subject interaction conceptually similar to the interaction observed in Thierry et al. (2009). We had 36 participants, while their sample size was 20. The reported partial eta squared (μ_p^2 = 0.112) for the mixed-effect (i.e., between-/within-participants) three-way interaction in Thierry et al. (2009) was associated with a 95% confidence interval whose lower bound was close to zero (0.004-0.273), which indicates imprecision and suggests that the effect they observed may range from very small to moderately large.

Free colour naming of colour circles by English speaking participants:

1) ***light blue:*** *blue n=21, light blue n=8, sky blue n=3, baby blue n=2, pale blue n=1, periwinkle n=1;*

2) ***dark blue:*** *blue n=29, dark blue n=4, deep blue n=1, royal blue n=1, turquoise n=1;*

3) ***light green:*** *green n=16, light green n=7, mint n=5, turquoise n=3, aquamarine n=1, lime n=1, matcha green n=1, sage green n=1, sage n=1;*

4) ***dark green:*** *green n=28, dark green n=5, forest green n=1, olive green n=1, olive n=1;*

5) ***pink:*** *pink n=27, light pink n=5, red n=2, rose n=1, salmon pink n=1;*

6) ***red:*** *pink n=10, red n=8, dark pink n=4, maroon n=4, magenta n=2, rose n=1, raspberry n=1, burgundy n=1, mauve n=1, purple n=1, purpley pink n=1, brown-red n=1, pinky maroon n=1;*

7) ***yellow:*** *yellow n=30, light yellow n=3, pale yellow n=2, green n=1;*

8) ***brown:*** *green n=11, brown n=11, dark green n=4, olive green n=2, olive n=1, hazel n=1, swamp n=1, taupe n=1, yellow n=1, khaki n=1.*

Supplementary Material 4 – Early negativities driven by differences in hue, saturation and luminance contrast (Experiment 3)

Initially, we fitted a model with the full set of fixed effects (deviancy*colour*lightness*saturation), but the step function from lmerTest R package indicated that hue (with two levels: red and green) contributed significantly to the model only on its own, rather than in interaction with the other three factors, which also failed to interact in a 3-way fashion with each other:

Deviancy:Hue:Saturation:Lightness, $F(1, 654.99) = 0.0174$, $p = 0.895$

Deviancy:Hue:Saturation, $F(1, 655.99) = 0.0198$, $p = 0.888$

Hue:Saturation:Lightness, $F(1, 656.99) = 0.2164$, $p = 0.642$

Hue:Saturation, $F(1, 657.99) = 0.8408$, $p = 0.359$

Deviancy:Hue:Lightness, $F(1, 658.99) = 1.1350$, $p = 0.287$

Deviancy:Hue, $F(1, 659.99) = 0.1456$, $p = 0.703$

Hue:Lightness, $F(1, 660.99) = 0.6421$, $p = 0.423$

Deviancy:Saturation:Lightness, $F(1, 662.00) = 3.091$, $p = 0.079$

A series of two-way interactions remained in the model, together with the fixed effect of hue:

Hue, $F(1, 663.00) = 9.706$, $p = 0.002$**

Deviancy:Saturation, $F(1, 663.00) = 7.301$, $p = 0.007$**

Deviancy:Lightness, F(1, 659.99) = 4.994, p = 0.026*

Saturation:Lightness, F(1, 660.99) = 6.427, p = 0.011*

The best-fitting model properties are listed below:

amplitude ~ deviancy + hue + saturation + lightness + (1 | participant) + deviancy:saturation + deviancy:lightness + saturation:lightness

	Amplitude (μV)		
Predictors	Estimates	95% CI	P
(Intercept)	1.94	[1.17, 2.71]	**<0.001**
Deviancy	-0.35	[(-0.56),(-0.14)]	**<0.001**
Hue	0.28	[0.10, 0.46]	**0.002**
Saturation	-0.17	[(-0.39), 0.04]	0.109
Lightness	-0.23	[(-0.45), (-0.02)]	**0.031**
Deviancy × Saturation	0.57	[0.16, 0.98]	**0.007**
Deviancy × Lightness	-0.47	[(-0.88), (-0.06)]	**0.026**
Saturation × Lightness	-0.55	[(-0.97), (-0.12)]	**0.011**
Random Effects			
σ^2	1.43		
$\tau_{00 \text{ participant}}$	3.75		
ICC	0.72		
$N_{\text{participant}}$	25		
Observations	695		
Marginal R^2 / Conditional R^2	0.019 / 0.729		

Post-hoc tests via emmeans package (using multivariate t distribution method for correction for multiple comparisons) revealed the following drivers for the observed interactions and the main effect of hue:

For saturation x lightness: Lower lightness was associated with lower amplitudes only when the stimulus was also desaturated (all other ps > 0.872):

higher saturation and lightness vs. desaturated lower lightness: 0.407, 95%CI [0.143, 0.672], t(663)= 3.015, p = 0.0139

desaturated of higher lightness vs. desaturated lower lightness: 0.508, 95%CI [0.175, 0.841], t(663)= 2.985, p = 0.0151

higher saturation and lower lightness - desaturated lower lightness: 0.4482, 95%CI [0.115, 0.7814], t(663) = 2.634, p = 0.0413.

For saturation x deviancy: Higher saturation standards elicited higher amplitudes (all other ps > 0.635):

Higher saturation standard vs. higher saturation deviant: 0.6363, 95%CI [0.393, 0.879], t(663) = 5.116, p <.0001

Higher saturation standard vs. desaturated standard: 0.4580, 95%CI [0.162, 0.754], t(663) = 3.043, p = 0.0128

Higher saturation standard vs. desaturated deviant: 0.5258, 95%CI [0.228, -0.824], t(663) = 3.464, p = 0.0030

For lightness x deviancy: Lower lightness deviants elicited lower amplitudes (all other ps > 0.781):

standard higher lightness - deviant lower lightness: 0.58541, 95%CI [0.287, 0.883], t(663) = 3.856, p< 0.001

deviant higher lightness - deviant lower lightness: 0.46846, 95% CI [0.173, 0.764], t(663) = 3.095, p = 0.0107

standard lower lightness - deviant lower lightness: 0.58713, 95% CI [0.254, 0.920], t(663) = 3.451, p = 0.0034

For hue: Green elicits lower amplitude than red:

green vs. red: -0.283, 95%CI [(-0.105), (-0.461)], t(663) = -3.115, p = 0.0019

Finally, we tested how stimuli with lower lightness or saturation sum up to evaluate the degree to which lightness and saturation are processed by separable neural resources. If this were the case, the predicted waveform for feature conjunctions (higher lightness and saturation or lower lightness and desaturated) should correspond to predictions made by averaging waveforms for single feature changes. Figure S5 below depicts predicted (full line) and observed (dotted line with grey 95% CIs) waveforms.

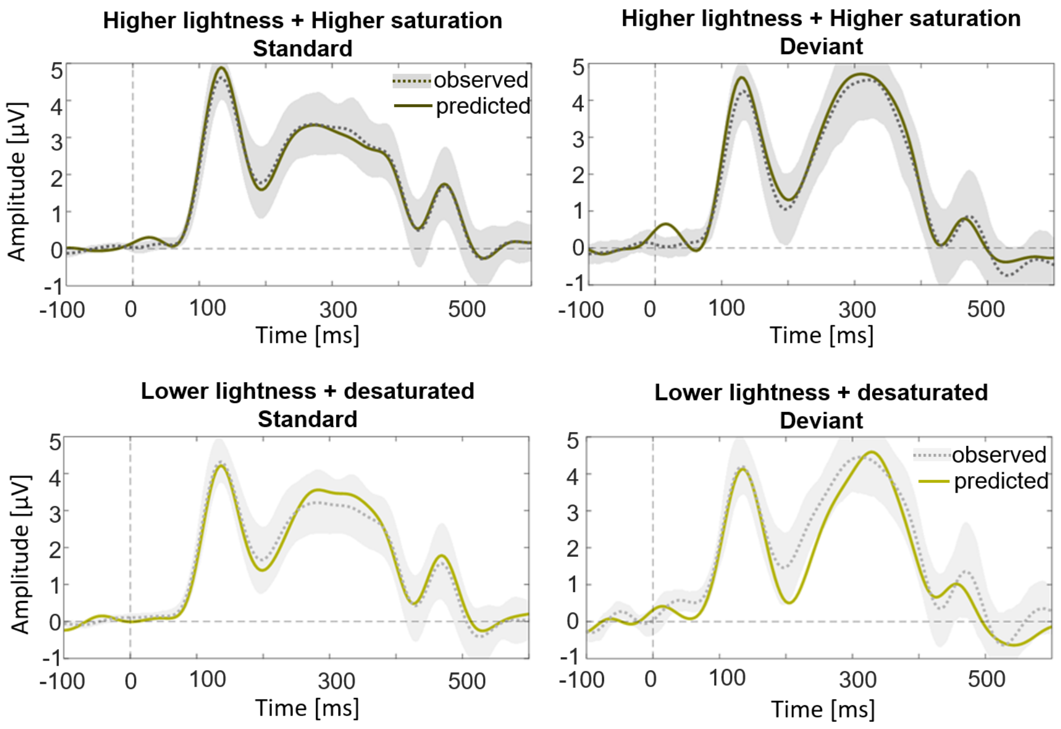

Figure S5. Visual evoked potentials (VEPs) observed in conditions that combined lightness and saturation changes, as well as waveforms predicted by averaging responses from VEPs elicited by changes in only one of the two features (lightness or saturation). Shaded grey area corresponds to a 95% between-subject CI for observed data.

As can be seen, the departure from the predicted waveform can be seen during the N1—P300 transition in the desaturated, lower lightness deviants. These stimuli were recorded in blocks with higher and more saturated standards, while single features, from which the predictions are derived, come from blocks in which the standards the participants were adapted to possessed only one of these two features. Observed responses to these stimuli in the N1—P300 window show amplitude that is reduced relative to what we predicted from single feature blocks. This is in line with shared neural resources between colour and luminance processing, which therefore can adapt more significantly (exhibiting repetition suppression).
